# Advancing Juvenile Spondyloarthritis: Closing Knowledge Gaps with New Axial JSpA Classification Criteria

**DOI:** 10.1007/s11926-025-01206-8

**Published:** 2025-11-18

**Authors:** Brittney N. Newby, Pamela F. Weiss

**Affiliations:** 1 Division of Pediatric Rheumatology, Children’s Hospital of Philadelphia, Philadelphia, PA USA; 2 Department of Pediatrics, Division of Rheumatology and Clinical Futures, Center for Clinical Epidemiology and Biostatistics, Perelman School of Medicine, Children’s Hospital of Philadelphia, University of Pennsylvania, Philadelphia, PA USA; 3 Roberts Center for Pediatric Research, CHOP, 2716 South Street, Room 11123, Philadelphia, PA 19146 USA

**Keywords:** Spondyloarthritis, Juvenile idiopathic arthritis, Clinical trial, Classification

## Abstract

**Purpose of Review:**

Until recently, the absence of validated, pediatric-specific classification criteria for juvenile spondyloarthritis (JSpA) limited targeted clinical trials evaluating treatment efficacy and advancements in understanding the natural history in pediatric-onset disease. There is an urgent need for efficacy and effectiveness studies in this understudied group.

**Recent Findings:**

Most children with JSpA continue to experience disease activity despite current therapies and generally have worse outcomes than those with other juvenile arthritis forms. Fewer than 20% achieve remission within five years of diagnosis. Axial involvement is a distinct manifestation warranting separate study and management, as it does not respond to conventional agents like methotrexate used for peripheral arthritis. Comparative effectiveness data are lacking, and no medications are FDA-approved specifically for “juvenile spondyloarthritis” or “juvenile ankylosing spondylitis.” The only FDA-approved therapy for enthesitis-related arthritis (ERA) is secukinumab. In 2024, pediatric classification criteria for axial disease in JSpA were published. These criteria include seven domains: MRI inflammation, MRI structural lesions, pain chronicity, pain pattern, pain location, stiffness, and genetic association. Imaging evidence of axial disease is necessary but not sufficient for classification.

**Summary:**

This review provides an overview of JSpA epidemiology, current and emerging classification criteria, and highlights the key features of the newly validated pediatric axial JSpA classification criteria.

## Introduction

JSpA encompasses a heterogeneous group of seronegative, immune-mediated diseases that begin in childhood and are strongly associated with the HLA-B27 allele. These conditions are characterized by enthesitis, peripheral arthritis, and axial involvement. They are also frequently accompanied by extra-musculoskeletal manifestations such as psoriasis, inflammatory bowel disease (IBD), and acute anterior uveitis.

Among all forms of juvenile idiopathic arthritis (JIA), an estimated 10–20% of patients exhibit features consistent with JSpA, including 10–15% with ERA and another ~ 5% with juvenile psoriatic arthritis (JPsA). The prevalence of JSpA (primarily ERA and JPsA) is estimated to range from 0.28 to 88 per 100,000 individuals, with higher rates observed in Asian countries [1]. However, prevalence varies across geographic regions, in part due to inconsistencies in classification and diagnostic approaches. Most patients present in early adolescence, with a peak onset around age 12. Compared to other JIA subtypes, JSpA shows a male predominance, though this is less pronounced than in adult-onset spondyloarthritis (SpA) [2].

The onset of JSpA is often insidious and varies significantly between individuals. Peripheral arthritis is more common than axial involvement in children and typically presents as asymmetric oligoarthritis of the lower extremities, most often affecting the ankles and hips. Foot involvement is also frequent, particularly in the tibiotalar and midfoot joints, though the metatarsophalangeal and subtalar joints may also be involved.

While ERA shares many features with adult SpA, key phenotypic differences exist. Pediatric-onset disease tends to involve more peripheral arthritis and less axial involvement at presentation [3–7]. In a multicenter inception cohort of children with ERA, 91% had peripheral arthritis and 75% at least one tender enthesis. Most had oligoarticular disease (75%), and approximately 60% were HLA-B27 positive, which is a lower proportion than seen in adult SpA [7]. Juvenile-onset disease is also associated with more aggressive hip involvement and is associated with a higher risk for hip arthroplasty [8]. In contrast, adult SpA more frequently presents with inflammatory back pain and predominant axial symptoms at onset.

Approximately 20% of patients with JSpA have axial disease at presentation, and up to 33% develop sacroillitis within a few years [5, 9–11]. The first study to assess the prevalence of sacroiliitis using magnetic resonance imaging (MRI) at the time of JSpA diagnosis was published in 2016 [5]. Among the 40 patients evaluated, 20% had sacroiliitis at presentation, most of which was asymptomatic. Of those with inflammatory sacroiliitis, 88% showed evidence of structural changes such as erosions or sclerosis. Despite this, only two patients met inflammatory back pain criteria as defined by the Assessment of Spondyloarthritis International Society (ASAS) criteria for inflammatory back pain, which are based on adult disease [5].

A 15-year longitudinal study from Norway found that 35% of ERA patients developed radiographic sacroiliitis, with most developing inflammatory back pain over time [12]. This disease trajectory differs somewhat from adult-onset undifferentiated SpA, in which around 60% of patients progress to ankylosing spondylitis within 10 years [13]. Notably, several studies suggest that axial disease in children is associated with worse long-term functional outcomes than adult-onset disease [14–16].

Diagnosis of axial involvement in children is particularly challenging. Only 38% of children with active sacroiliitis report symptoms such as back pain or tenderness [5]. Moreover, clinical examination of the sacroiliac joints in pediatric patients is unreliable, with significant inter-provider variability and poor correlation with inflammation detected by MRI [5, 17, 18]. These limitations highlight the inadequacy of symptom-based screening in children and underscore the importance of imaging in the early identification of axial disease [5, 10, 18].

## Challenges in JSpA Research with a Focus on Axial Disease

Conducting clinical trials in JSpA presents numerous challenges, including limited feasibility due to small sample sizes, as well as the multisystemic nature and heterogeneous clinical presentation of the disease, which complicate classification. Furthermore, the absence of JSpA and pediatric-specific axial outcome measures limits the ability to accurately assess the effectiveness of biologic and synthetic DMARD therapies. Traditional clinical trial designs are often impractical in pediatric rheumatology, and the availability of effective treatments raises ethical concerns around the use of placebo arms. As a result, many studies rely on extrapolation from adult data or are based on very small pediatric cohorts—approaches that may fail to account for critical features unique to pediatric disease. Pediatric researchers are beginning to adopt innovative trial designs to address these challenges. A notable example is the BACK-OFF JSpA trial, which incorporates patient and parent stakeholders in trial design to develop a pragmatic approach to address important questions important to both patients and clinicians alike [19].

Until recently, the lack of standardized classification criteria for study inclusion has significantly hampered efforts to design focused and inclusive trials in both adults and children. Most existing studies have targeted either peripheral or axial arthritis in isolation, often relegating hallmark features such as enthesitis, dactylitis, and extra-articular manifestations to secondary outcomes. Since 1999, eight biologic medications have been approved for use in polyarticular (≥ 5 joints affected by arthritis during the first six months of disease) JIA: etanercept (1999), adalimumab (2014), abatacept (2008), tocilizumab (2013), golimumab (2020), tofacitinib (2020), sarilumab (2024), and upadacitinib (2024). However, only about one-third of children with JSpA present with polyarticular disease. In pivotal phase III and IV trials for these agents, children with JSpA were either a small subset or entirely excluded. This selective inclusion has contributed to a substantial evidence gap, leaving much of the JSpA population underrepresented.

## Limitations of Prior Classification Criteria in Juvenile SpA

Although JSpA is often used as an umbrella term to describe a diverse group of related conditions, this terminology is not commonly adopted in routine pediatric rheumatology practice, complicating direct comparisons between pediatric and adult-onset disease. The most widely used classification system, the International League of Associations for Rheumatology (ILAR) criteria [20], categorizes some forms of JSpA into distinct subtypes such as ERA and JPsA. Other related conditions that don’t fulfill either the ERA or PsA criteria, including juvenile-onset ankylosing spondylitis, reactive arthritis, and IBD-associated arthritis, are grouped into an “undifferentiated arthritis” category. In clinical practice, many children exhibit overlapping clinical features that do not fit neatly into the mutually exclusive ILAR categories. As a result, they are frequently labeled as “undifferentiated,” which can limit their inclusion in research studies and restrict access to targeted therapies that might otherwise be beneficial. Moreover, up to one-third of children with axial disease alone do not meet any ILAR criteria due to the lack of peripheral disease, excluding this important group of patients [21].

Prior studies have raised concerns about the ILAR classification criteria’s ability to accurately identify patients with JSpA, particularly those with isolated axial involvement or phenotypes with overlapping features with other JIA categories [22]. For example, a comparative study evaluating the ILAR criteria against other proposed classification systems reported a sensitivity of 79.6% and a specificity of 74.3% [23]. A 2022 study looking at the ability of the ILAR criteria to identify ERA showed only moderate sensitivity at diagnosis (74%), which improved to 82.4% at follow-up [24], while specificity remained high at 100%. Importantly, the ILAR system does not incorporate advanced imaging findings, which are tremendously important for early and accurate detection of JSpA [25].

To address these limitations and improve the classification of juvenile idiopathic arthritis (JIA), the Pediatric Rheumatology International Trials Organization (PRINTO) proposed a new set of provisional criteria [26]. Developed through a multi-phase process involving Delphi rounds and a nominal group consensus conference, the PRINTO framework introduces updated categories: systemic JIA, RF-positive JIA, enthesitis/spondylitis-related JIA, early-onset ANA-positive JIA, and other JIA. Key differences from the ILAR criteria include raising the age-of-onset threshold from under 16 to under 18 years, removing the requirement for arthritis in systemic JIA, and eliminating joint count as a criterion for subtyping. Under the PRINTO criteria, patients with JSpA are typically categorized within the enthesitis/spondylitis-related JIA category. This includes patients who have peripheral arthritis and enthesitis; or arthritis or enthesitis plus more than 3 months of inflammatory back pain and sacrolillitis on imaging; or arthritis or enthesitis plus 2 related clinical features including sacroiliac tenderness, inflammatory back pain, HLA B27, acute anterior uveitis or history of SpA in first degree relative.

Despite these improvements, both the ILAR and PRINTO classification systems still miss a significant portion of patients with axial JSpA. Recent data suggest that between one-quarter to one-third of such patients remain uncaptured by these criteria, and approximately 20% do not meet any current ILAR or PRINTO category [21]. Two large prospective cohorts, the Canadian ReACCH-Out cohort and the British Childhood Arthritis Prospective Study (CAPS), compared the ILAR and PRINTO provisional criteria. Under the ILAR framework, 12–21% of each cohort was classified as undifferentiated. In contrast, applying the PRINTO criteria resulted in 63–70% of patients falling into the “other JIA” category. These findings suggest that, while the PRINTO criteria represent progress, they may still result in large proportions of patients being placed into broad, non-specific categories [27, 28].

## Evolution of Spondyloarthritis Classification in Adults

The classification criteria for adult SpA have undergone significant evolution over the past six decades. Early efforts, including the 1961 New York Criteria and the 1966 Rome Criteria, centered on the clinical and radiographic features of ankylosing spondylitis (AS). The 1984 modified New York Criteria became the widely accepted standard, emphasizing late-stage sacroiliitis detectable on plain radiographs, along with clinical features such as inflammatory back pain, restricted chest expansion, and limited spinal mobility. While these criteria demonstrated high specificity for established ankylosing spondylitis, they lacked sensitivity for identifying early or non-radiographic forms of the disease [29, 30].

In the early 1990 s, two alternative classification systems, the Amor criteria and the European Spondyloarthropathy Study Group (ESSG) criteria, were introduced to improve the classification of spondyloarthritis. These criteria attempted to expand the clinical and lab parameters used in the diagnosis of SpA. Both were developed before the widespread use of MRI and HLA-B27 testing. As a result, they lacked sensitivity for detecting early or non-radiographic disease and often failed to identify patients without classic radiographic findings, leading to under-recognition of disease. Moreover, these criteria did not differentiate between axial and peripheral disease manifestations, limiting their utility in both clinical and research settings. Ultimately, the Amor and ESSG criteria were supplanted by the The Assessment of SpondyloArthritis International Society (ASAS) classification criteria introduced in 2009, which offered improved sensitivity and specificity, particularly for non-radiographic disease.

ASAS introduced separate classification criteria for axial and peripheral SpA [31–33]. These classification criteria were designed for use in clinical research to recruit relatively homogeneous patient populations and were not intended to serve as diagnostic tools in routine clinical practice. To meet ASAS criteria for axial SpA, patients must have at least three months of chronic back pain with onset before age 45. From there, classification can occur through one of two entry arms: the clinical arm or the imaging arm. Under the clinical arm, patients must be HLA-B27 positive and exhibit at least two additional features suggestive of SpA, such as peripheral arthritis, dactylitis, enthesitis, a family history of SpA, inflammatory bowel disease, psoriasis, a positive response to NSAIDs, or elevated C-reactive protein. Under the imaging arm, patients must have evidence of sacroiliitis—either on plain radiographs using the modified New York criteria [29] or on MRI per ASAS definitions [34–36] plus at least one clinical feature of SpA. In adults, the overall sensitivity and specificity of the axial ASAS criteria are 82.9% and 84.4%, respectively [31]. However, when assessed separately, the imaging criteria offers higher specificity (sensitivity: 66.2%, specificity: 97.3%) compared to the clinical criteria (sensitivity: 56.6%, specificity: 83.3%). In contrast, the ASAS classification criteria for peripheral SpA focus on patients presenting with arthritis, enthesitis, or dactylitis in combination with other typical SpA features. These criteria demonstrate a sensitivity of 77.8% and a specificity of 82.2% [33].

Since their development, the ASAS criteria have been widely adopted in clinical trials and have supported the regulatory approval of several therapies for SpA. However, concerns have emerged regarding the potential misuse in clinical practice, particularly the risk of overdiagnosis when applied outside of research settings [37]. In response, ASAS and the Spondyloarthritis Research and Treatment Network (SPARTAN) launched the Classification of Axial Spondyloarthritis Inception Cohort (CLASSIC) study to re-evaluate and refine the specificity of these criteria, an effort that remains ongoing. This has also led to an international consensus on optimal MRI protocols for evaluating sacroiliac joints in axial SpA, recommending the use of fluid-sensitive sequences in semicoronal and axial planes, fat-sensitive T1-weighted sequences, and erosion-sensitive sequences in the semicoronal orientation [38].

Unfortunately, classification criteria developed for adult SpA have not translated well to children, likely due to differences in disease phenotype and age-related clinical manifestations. Adult criteria tend to have lower sensitivity when applied to JSpA, particularly for axial disease. For example, the ASAS axial SpA criteria have shown sensitivities as low as 21–35% in ERA cohorts, although specificity remains high [23, 24, 39]. Although specificity is prioritized, such low sensitivity is inadequate for identifying a substantial proportion of affected children in both clinical and research settings. This limitation is not surprising as inflammatory back pain, a required entry criterion for ASAS axial SpA, is often absent or difficult to assess in children. Similar evaluations of the Amor and ESSG criteria in pediatric populations have shown somewhat improved sensitivity, but they still fall short in detecting non-radiographic disease [23, 24]. This underscored the pressing need for pediatric-specific classification criteria that more accurately reflect the unique features of axial disease in JSpA.

## Charting New Territory: A New Era of Classification in Axial Juvenile Spondyloarthritis

In 2024, the field of pediatric rheumatology achieved a major milestone with the publication of the first validated classification criteria for axial disease in youth with JSpA. This pivotal advancement addresses a long-standing gap by providing standardized, evidence-based criteria to reliably identify children with axial JSpA and facilitate their inclusion in pediatric-specific clinical studies. The effort was led by an international team comprising expert pediatric rheumatologists, experts in multicriteria decision analysis, a central imaging review panel, an independent validation team, and dedicated data management specialists. The criteria are intended for use in patients less than 18 years of age with a confirmed clinical diagnosis of JSpA.

**Fig. 1:**
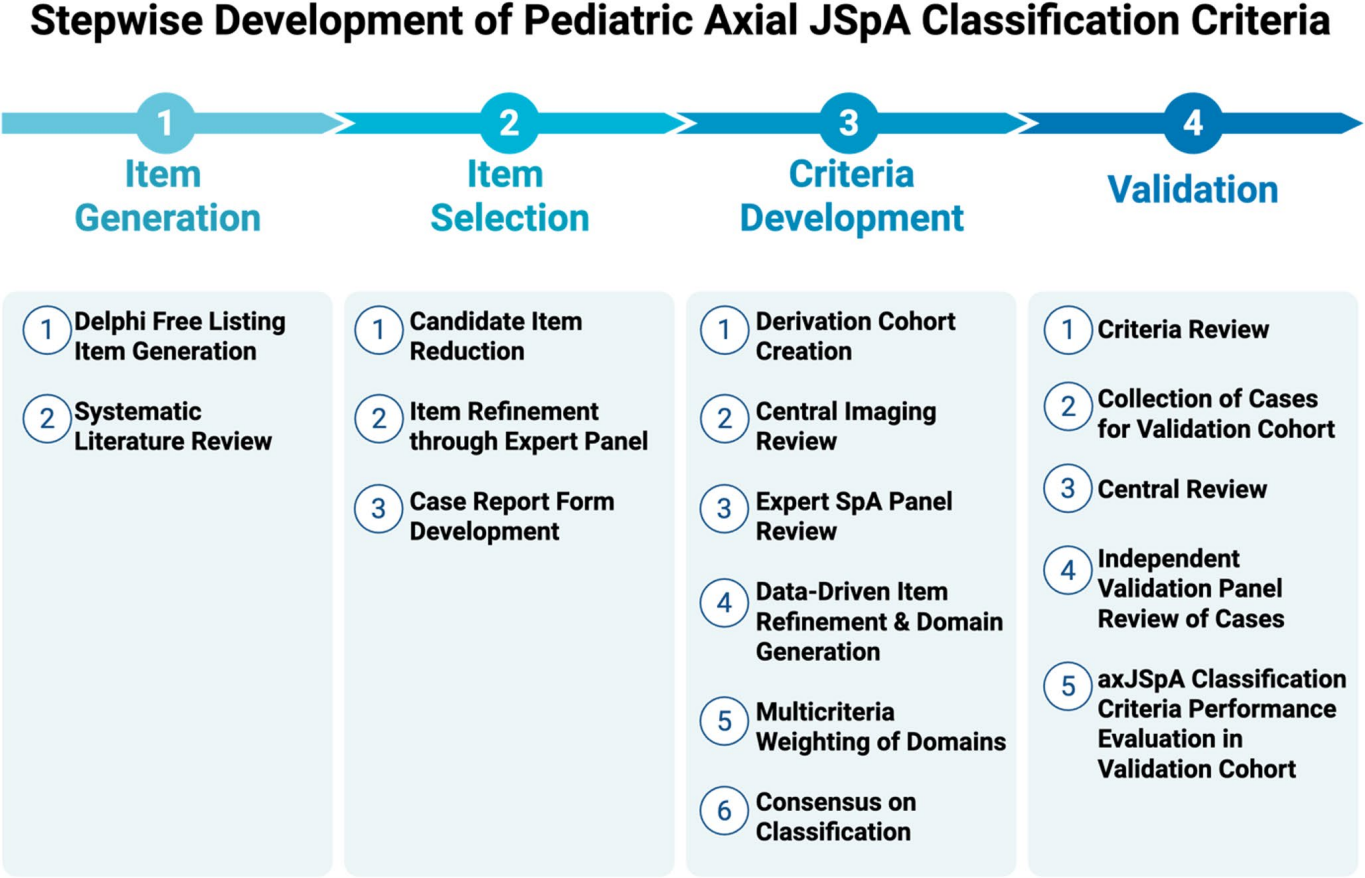
Stepwise Development of Pediatric Axial JSpA Classification Criteria. Created in BioRender. Newby, B. (2025) https://BioRender.com/kdpgql0

Using a structured, multiphase approach as shown in Fig. 1, the project began with a free-listing exercise and comprehensive literature review, generating 108 candidate items for classification. In Phase 2, these items were then evaluated and ranked by the same group (> 100 international physicians) that participated in the free-listing exercise based on their likelihood of indicating axial disease in children. Items deemed redundant or of low-influence were removed. The remaining items were grouped into relevant domains by the clinical expert panel (Fig. 1).

In the next phase, the team assembled a cross-sectional derivation cohort consisting of 303 patients with a clinical diagnosis of JSpA (Fig. 1). This cohort included cases (patients with axial arthritis) and controls (patients with JSpA and other etiologies for axial symptoms). All participants underwent a dedicated pelvic MRI as part of the diagnostic evaluation and clinical data were collected on standardized electronic case report forms. Imaging was centrally reviewed, including lesion-based assessments of both inflammatory and structural lesions, and a global assessment, “Are these inflammatory and/or structural lesions typical of axial SpA?”.

As part of this effort, the group also developed a consensus definition of pediatric-specific imaging features indicative of typical axial involvement on radiograph and MRI [40, 41]. Each participant’s case report, including clinical and central imaging data, was independently reviewed by three SpA experts who rated the likelihood of axial disease. Expert consensus was achieved for 251 patients (82.8%), including 118 cases with axial disease and 133 non-axial cases; the remaining 52 participant cases were excluded due to a lack of consensus.

The next step was to refine and weight the classification items using a data-driven approach. Items were leveled within each domain based on their strength of association with expert-confirmed axial disease. The expert panel iteratively reviewed and adjusted the item levels as needed. Once finalized, a multicriteria decision analysis was used to assign weights to each domain and item. The final set of criteria included seven domains: active inflammation on MRI, image-confirmed structural lesions, chronicity of pain, pain pattern, pain location, stiffness, and genetic association (Table 1). The final scoring algorithm had a maximum score of 100. A score of 55 or higher indicates classification of axJSpA. Importantly, while MRI evidence of axial inflammation or damage was required, it was not sufficient on its own, clinical features also had to be present for classification.

**Table 1:** Weighted scoring for axial juvenile spondyloarthritis classification criteria

Clinical Domains & Criteria	Weight	Maximum Domain Score
**Imaging: Active Inflammation**		
No active sacroiliitis on SIJ MRI	0	
Clear evidence of active sacroiliitis on SIJ MRI	23	**23**
**Imaging: Structural Lesions**		
No evidence of structural lesions of the SIJs on MRI	0	
Evidence of sacroiliitis on radiograph^#^	13	**23**
Evidence of structural lesions of the SIJs on MRI	23	
**Pain Chronicity**		
Pain < 4 days per week OR Pain < 6 weeks	0	
Pain > 4 days per week for ≥ 6 but < 12 weeks	6	**9**
Pain > 4 days per week for ≥ 12 weeks	9	
**Pain Pattern**		
No Pain Pattern	0	
Awakens patient second half of night or insidious onset	6	**13**
Moderate to total relief with NSAID	10	
Improves with activity	13	
**Pain Location**		
No back, groin, hip, or buttock pain	0	
Lumbar spine pain (patient-reported)	6	**12**
Sacroiliac pain with deep palpation or elicited using clinical maneuver^†^‡ or Groin/Hip pain (patient-reported)	11	
Sacral/buttock pain (patient-reported)	12	
**Morning Stiffness**		
No stiffness or stiffness < 15 min	0	**9**
Stiffness ≥ 15 min	9	
**Genetics**		
No family history OR HLA-B27 unknown/negative	0	
1 st degree relative with SpA or HLA-B27 associated AAU	8	**11**
HLA B27 Positive	11	
Axial Juvenile Spondyloarthritis if Score ≥ 55		**100**

To be classified as axial JSpA, patient must have 55 or greater score. Levels within each domain are mutually exclusive. Highest level achieved within each domain contributes to overall score. # only applicable in absence of pelvic MRI. † clinical maneuvers commonly used to assess sacroiliac joint involvement include the FABER (Flexion, Abduction, external Rotation), Mennell, and Gaenslen tests. In the FABER test, pain elicited in the contralateral sacroiliac joint is considered a positive finding. Mennell’s maneuver produces SI joint pain when downward pressure is applied with the patient prone, leg straight, and hip extended. In gaenslen’s test, the patient Lies supine with one hip and knee flexed while the contralateral hip is hyperextended by applying downward force, reproducing SI joint pain on the side being extended

The criteria were then tested in an independent validation cohort of 219 international patients with JSpA and reviewed by a separate panel of five juvenile arthritis experts (Fig. 1). Agreement on the presence/absence of axial disease was achieved in 89.5% of cases. Within the validation data set, the axJSpA final criteria demonstrated a specificity of 97.5%, sensitivity of 64.3%, and AUROC of 0.81. Importantly, the axJSpA classification criteria outperformed all previously existing criteria, including ASAS axial SpA, ASAS peripheral SpA, ESSG, ILAR ERA, and the ILAR ERA sacroiliitis definition.

## Conclusions

The recent development of validated classification criteria for axial JSpA marks a pivotal advancement in pediatric rheumatology. For decades, the absence of pediatric-specific criteria has limited our ability to promptly identify affected children, enroll them in clinical trials, and generate high-quality evidence to guide treatment decisions. While the ILAR and PRINTO frameworks have provided an important foundation for classifying juvenile arthritis, they have consistently fallen short in capturing the full spectrum of JSpA, particularly in cases with isolated axial disease. Similarly, adult SpA classification systems translate poorly to pediatric populations.

The newly established axJSpA criteria begin to address these longstanding gaps by offering a rigorous, evidence-based approach to classifying axial involvement in children with JSpA. Their integration of both clinical and imaging features prioritizes diagnostic specificity and reflects the unique presentation of pediatric disease. With these criteria now available, the juvenile spondyloarthritis community is better equipped to conduct focused clinical trials, refine disease phenotyping, and advance precision medicine approaches in JSpA. The implementation of these criteria represents a foundational step toward improving outcomes for children with JSpA, while ongoing refinement and validation will be critical to supporting tailored therapies and optimizing long-term outcomes for children with axial disease.

## Key References

- Weiss PF, Brandon TG, Aggarwal A, Burgos-Vargas R, Colbert RA, Horneff G, et al. Classification Criteria for Axial Disease in Youth With Juvenile Spondyloarthritis. Arthritis Rheumatol. 2024;76 [12]:1797 − 808.
  - This article establishes the first validated classification criteria for axial disease in youth with juvenile SpA, addressing a major gap that has long limited research and clinical trial design in this population. These criteria will enable more consistent identification of affected patients and advance studies on treatment and outcomes.
- Adrovic A, Sezen M, Barut K, Sahin S, Acikel C, Demirkaya E, et al. The performance of classification criteria for juvenile spondyloarthropathies. Rheumatol Int. 2017;37 [12]:2013-8.
  - This study evaluates how well existing adult and pediatric classification criteria perform in identifying juvenile SpA, highlighting their strengths and limitations. The findings underscore the need for pediatric-specific criteria to improve diagnosis and research in this population.
- Maksymowych WP, Lambert RG, Ostergaard M, Pedersen SJ, Machado PM, Weber U, et al. MRI lesions in the sacroiliac joints of patients with spondyloarthritis: an update of definitions and validation by the ASAS MRI working group. Ann Rheum Dis. 2019;78 [11]:1550-8.
  - This article refines and validates MRI lesion definitions for sacroiliac joints in SpA, providing standardized imaging criteria to improve diagnostic accuracy and consistency. These updates are critical for both clinical practice and research, ensuring more reliable assessment of disease activity and progression.
- Dubreuil M, Deodhar AA. Axial spondyloarthritis classification criteria: the debate continues. Curr Opin Rheumatol. 2017;29 [4]:317 − 22.
  - This article reviews ongoing controversies surrounding axial SpA classification criteria, particularly the balance between sensitivity and specificity and their implications for diagnosis and research. It emphasizes the need for continued refinement to optimize both clinical care and trial enrollment.
- Weiss PF, Brandon TG, Lambert RG, Biko DM, Chauvin NA, Francavilla ML, et al. Data-Driven Magnetic Resonance Imaging Definitions for Active and Structural Sacroiliac Joint Lesions in Juvenile Spondyloarthritis Typical of Axial Disease: A Cross-Sectional International Study. Arthritis Care Res (Hoboken). 2023;75 [6]:1220-7.
  - This article establishes data-driven MRI definitions for active and structural sacroiliac joint lesions in juvenile spondyloarthritis, tailored specifically to axial disease in youth. These standardized definitions enhance diagnostic precision and provide a foundation for future research and clinical trials.

## Data Availability

No datasets were generated or analysed during the current study.
